# Reply to the letter to the editor by J. Hissbach, S. Zimmermann & W. Hampe

**DOI:** 10.3205/zma001092

**Published:** 2017-05-15

**Authors:** Iris Kesternich, Heiner Schumacher, Joachim Winter, Martin R. Fischer, Matthias Holzer

**Affiliations:** 1 Universität Leuven, Fachbereich Ökonomie, Leuven, Belgien; 2 Universität München, Volkswirtschaftliche Fakultät, Seminar für Empirische Wirtschaftsforschung, München, Deutschland; 3 Klinikum der Universität München, Institut für Didaktik und Ausbildungsforschung in der Medizin, München, Deutschland

## Letter to the Editor

We appreciate the letter to the editor, “Student selection cannot solve the lack of rural and family doctors.” by J. Hissbach, S. Zimmermann and W. Hampe [1] concerning our article “Student characteristics, professional preferences, and admission to medical school.” GMS J Med Educ. 2017;34(1):Doc5 [2].

For the bivariate probit model that we used for the multiple regression analysis, the specification of a pseudo-R² is unusual. The quality of our model can be characterized by a Chi-squared test which rejects the null hypothesis that all coefficients of the model are jointly not all equal to zero with a p-value of 0.029. Therefore we consider our results to be meaningful – with the methodological caveats made in the article concerning the representativeness of the sample and the fact that we can only report the self-assessment of the students and not their actual professional choice.

In our sample, 22% of students who aspire to work as general practitioners in the countryside (n=16) have been waiting for at least one semester but only 18% of the future non-countryside doctors had to wait. The average number of waiting semesters was 2.12 among all 73 participants with the wish to practise in the countryside compared to 1.40 among all future non-rural doctors. If one only takes into account the students who have at least one waiting semester, the average number of waiting semesters is 7.96 (“non-countryside”) versus 9.69 (“countryside”). Thus, the length of waiting time in our sample is well below the average waiting period of 7 years [3].

Students from the waiting quota have a lower success rate than the students admitted through other quotas – as noted in our article (see [4]). It would certainly be of great interest to examine the students with waiting semesters and the desire to work in the countryside in further studies and to provide them with specific courses of study in the first years in order to better reflect their different prior knowledge and their greater practical experience.

Indeed, our dataset does not contain information about subjects’ voluntary social engagement. The goal of our discussion was to promote the opportunity to screen applicants based on their voluntary engagement. In a recent research project, we show that voluntary social engagement is informative about personal preferences [5]. Therefore, the selection based on résumé content seems to be a promising method to alleviate the shortage of general practitioners and country doctors. At this point, it is an open question whether this approach is successful.

Of great importance is that curricula sufficiently reflect such topics as outpatient care in urban and rural areas in the general and primary care sectors. The institutional conditions in Germany are currently better than ever before, thanks to chairs for family medicine at almost all medical faculties and extensive networks of training practices.

More generally, the question arises whether it is possible to reliably predict the later career plans of the graduates already at the time of admission. Criteria for student selection derived from the needs of the health care system and the preferences of current students are certainly not without problems. In our view, however, they represent a potential opportunity to increase the proportion of graduates of medical students who are pursuing a medical practice in the countryside in addition to incentives which only take effect after the end of the study. Whether this objective, which is obviously motivated politically, can be achieved, must be shown by future studies that examine the connection between different conceivable selection criteria and the actual professional choice decisions of the future physicians.

## Competing interests

The authors declare that they have no competing interests.
